# Characterisation of Formaggella della Valle di Scalve Cheese Produced from Cows Reared in Valley Floor Stall or in Mountain Pasture: Fatty Acids Profile and Sensory Properties

**DOI:** 10.3390/foods9040383

**Published:** 2020-03-26

**Authors:** Paolo Formaggioni, Massimo Malacarne, Piero Franceschi, Valentina Zucchelli, Michele Faccia, Giovanna Battelli, Milena Brasca, Andrea Summer

**Affiliations:** 1 Department of Veterinary Science, University of Parma, Via del Taglio 10, I-43126 Parma, Italy; paolo.formaggioni@unipr.it (P.F.); andrea.summer@unipr.it (A.S.); 2 Veterinary Freelance, Via Monte Grappa 7, I-24020 Vilminore di Scalve (BG), Italy; valentina.zucchelli78@gmail.com; 3 Department of Soil, Plant and Food Sciences, University of Bari, Via Amendola 165/A, 70125 Bari, Italy; michele.faccia@uniba.it; 4 Consiglio Nazionale delle Ricerche, Istituto di Scienze delle Produzioni Alimentari, UT di Milano, Via Celoria 2, IT-20133 Milano, Italy; giovanna.battelli@ispa.cnr.it (G.B.); milena.brasca@ispa.cnr.it (M.B.)

**Keywords:** cheese quality, mountain cheese, fatty acid profile, volatile organic compounds, sensory properties

## Abstract

An important problem in mountain areas is the abandonment of pasture. This trend can be combated by the valorisation of typical dairy products, such as “Formaggella della Valle di Scalve”, a semi-cooked traditional cheese made from whole milk in a mountain area in Italy. The aim of the present research was to compare the fatty acid (FA) profile and the sensory properties of this cheese as manufactured under different conditions: i) from the milk of cows grazing on mountain or valley pasture or fed indoors; ii) from the milk of cows fed hay or fed silage. In the first case, five cheesemaking trials were conducted during two years for each of the following situations: mountain pasture (A); pasture at the bottom of the valley (P) (about 1000m asl); stall (S). In the second case, three cheesemaking trials were conducted for each of the following situations: cows fed silage (I); cows fed hay (F). S cheese was richer in medium-chain FAs, while long-chain FAs were higher in P and A cheeses. On the other hand, long chain fatty acids (LCFA) were more abundant in P and A cheeses than in S. In general, MUFA, PUFA and, consequently, total unsaturated FA (UFA), were significantly higher in the P and A cheeses than S (UFA: 36.55 and 38.34, respectively, vs. 31.13; *p* < 0.001), while SFA showed higher values in S (68.85 vs. 63.41 and 61.68 in P and A, respectively; *p* < 0.001). Conjugated linoleic acid isomers (CLA) were more represented in the P and A samples (1.86 in P and 1.52 in A, vs. 0.80 in S; *p* < 0.001); Omega 3 fatty acids, and in particular α-linolenic acid, were more abundant in P than in S cheese. In winter, the I sample (silage) presented higher percentages of myristic (C14), myristoleic (C14:1) and omega 6 acids, whereas F cheese (hay) contained higher concentrations of CLA. The triangular test of sensory analysis showed that, in general, F cheeses were judged as “sweeter” than I, with aromatic profiles characterized by higher content of 2- butanol and ethyl capronate.

## 1. Introduction

One of the main problems in the mountain areas in Italy is the abandonment of marginal portions of the territory, widely utilised in the past for traditional activities (agriculture, livestock, forestry). In the specific case of Valle di Scalve (Lombardy Region, province of Bergamo), the causes of the phenomenon are to be found in the social, cultural and economic changes that affected mountain areas in the second post-war period. Agricultural and forestry activities, which were previously the basis of the self-consumption economy characterizing the existence of each family, were progressively supplanted first by industrial activity and successively by that of the tertiary sector. The national and local government continue to put in place interventions aiming to encourage the resumption of active management of the mountain pastures in this area. A common strategy is focusing on the valorisation of typical dairy products, such as Formaggella della Valle di Scalve cheese. This product is made from raw whole milk added to with a natural milk-starter (milk of the day before, left to ferment overnight) and coagulated with commercial rennet. It is a semi-cooked cheese weighing about 2 kg, ripened from 20 days up to 3 months. It is cylindrical in shape, with about a 20 cm diameter and a 6 cm heel, and bears the impressed brand (a stylised bear, the symbol of the Valley) of the cooperative company at which it is manufactured (Latteria Sociale Montana). The average gross composition at 30 days’ ripening is shown in Table 1.

Formaggella della Valle di Scalve is manufactured according to traditional technology, descending from the multigenerational experience of the local cheese makers. Milk is produced in valley floor stall (indoor) during winter and on mountain pastures during summer, even though some farmers remain at the valley stall for the whole year. Even though the traditional breeding system of dairy cattle involves the use of high-altitude pastures during summer, the share of farms keeping animals in the stable all year long with a hay-based diet is increasing [1]. Pastures are still widespread in mountain environments and, apart from their valuable contribution to livestock production, they contribute the promotion of local tourism, biodiversity conservation, maintenance of landscapes and mitigation of pollution [2]. In this context, the role of mountain farms in preserving ecological equilibrium and historic traditions, and in maintaining the landscape for protection against hydro-geological disorder, has been widely recognized [2]. 

Unfortunately, mountain farms are poorly competitive and have high production costs due to the unfavourable natural conditions. Therefore, promoting mountain products is an appropriate strategy for generating wealth and preventing the abandoning of mountain farming. Consumers’ perception of the mountain food products is good since they are commonly linked to concepts such as tastiness, healthiness, wholesomeness, animal welfare, history and local culture [2,3]. Mountain cheeses often have unique sensory properties that are deeply connected to the environmental conditions of milk production [4]. In particular, milk native microbiota deriving from the farm and cheesemaking environment are known to play a key role in determining the organoleptic characteristics [5]; for this reason, raw milk cheeses tend to develop a more intense flavour than pasteurised milk cheeses [3,6].

Besides the role of microbiota, animal feeding can modify the composition and rennet-coagulation properties of the milk [7,8], and the characteristics of the cheese [9]. In particular, the nature of the pasture is responsible for changes in cheese colour and aroma according to the type of forage fed to animals, as frequently reported by farmers and cheese makers. Recent studies conducted on various cheese varieties [10] have demonstrated the influence of forage preservation (e.g., grass versus hay [11], silage versus hay [12]) and the botanical composition of dry forage [13]. Other studies have been conducted to identify, quantify, and understand the effects of forage type (e.g., maize silage, hay, grass silage, pasture) [14,15]. According to several studies, the sensory properties of the cheeses made from “pasture milk” can reflect the characteristics of the fresh pasture plants [16,17] and of the natural microbiota from both the animal and the environment [18]. For certain, the diet of dairy cattle influences the colour [19], in connection with seasonal variations of the concentration of β-carotene in milk [20]. In general, consumption of green forage increases the β-carotene content of milk and cheese [21]. There is also evidence about the desirable effect of grasslands on the distinctive flavour of dairy products [19]. Kilcawley et al. [22], in a review, have recently analysed the factors influencing the sensory characteristics of bovine milk and cheese from grass-based versus non-grass-based milk production systems. All the sensory properties—odour, aroma, taste and texture—of cheeses made with pasture-derived milk may be different from those made with dry forage milk [1,23]. Compared with hay, pasture is known to provide a less firm, and more creamy, texture [14,15,24]. These characteristics are also modified during ripening because of the different enzymatic processes, including proteolysis and lipolysis, which also play a key role in aroma development [1]. Carpino et al. [19], found that the cheeses from pasture-fed cows had a significantly more floral and greener odour, as measured by quantitative descriptive analysis. 

The type of forage deeply influences the milk fatty acid (FA) composition, both as to the type and the proportion of the compounds [25]. Fresh green forage (having higher levels of polyunsaturated fatty acids relative to silage) allows the production of milk with higher content of these type of FA [26] (especially *cis*-9-C18:1, *trans*-11-C18:1, *cis*-9,*trans*-11-CLA, and C18:3 n-3; CLA: Conjugated Linoleic Acids) and poorer in saturated FA (in particular C16:0, C14:0, and C12:0 [27,28]) compared with cows fed with preserved forages and concentrates [14]. Many studies have been carried out on this topic, particularly with regard to the effects of different diets on the content of unsaturated long-chain fatty acids, such as linolenic acid and conjugated linoleic acid [26,29,30,31]. These acids are claimed to have positive effects on human health, and a number of reports are available in the literature about ways of naturally increasing, through cow feeding, their content in milk [31]. In this regard, attention has been paid to the level of polyunsaturated fatty acids (PUFA), especially conjugated linoleic acid (CLA), in milk fat [29]. Although most of the CLA of milk fat are synthesized in the mammary gland, some of them represent an intermediate of ruminal biohydrogenation of linoleic acid [32]. For this reason, pasture feeding, yielding higher levels of PUFA, causes a higher CLA content of milk fat compared to feeding conserved forage [29].

As a consequence of this, consumers have a growing interest in mountain dairy products, which can be considered as functional foods in the proper sense. In fact, from a nutritional point of view, pasture-derived dairy products seem particularly interesting [1]: the FA profile is favourable to human health, being characterised by a higher content of PUFA. In effect, the haymaking process, i.e., mechanical damage to plant tissues combined with air access, causes extensive oxidation of PUFA [1]. In addition, CLA can beneficially modulate several important physiological functions [33,34]. Moreover, the traditional feeding system can also transmit biologically active molecules (such as β-carotene), which have beneficial effects on human health as powerful antioxidants protecting against oxidative stress [35].

The aim of the study was to compare the quality characteristics of Formaggella della Valle di Scalve produced under different conditions: a) in summer, from milk of cows grazing on mountain pasture, in valley floor pasture (1000 m quota) or fed indoors with a hay-based diet; b) in winter, from milk of cows fed hay or fed silage.

## 2. Materials and Methods

### 2.1. Experimental Design and Sampling Procedure

Summer phase. Five cheesemaking trials were conducted in summer over two years for each of the following experimental situations: stall (S) (indoor, at valley floor, about 1000 m asl; cows were fed permanent meadow hay and grass); pasture (P) (at the bottom of the valley, about 1000 m asl); mountain pasture (A). This latter is defined as the grazing of cattle in the high mountains, from altitudes greater than 1000 m up to 2300–2500 m, carried out from late May to mid-September.

Winter phase. Three cheesemaking trials were conducted for each of the following situations: cows reared in stall fed permanent meadow hay silage (I); cows reared in stall fed permanent meadow hay without silage (F). The winter tests began after the adaptation phase, when cows returned to the barn from the mountain pasture or pasture at 1000 m. 

In both cases, the groups of cattle were, as far as possible, homogeneous by breed, lactation stage and deliveries of the cows. Just before cheesemaking, a milk sample was taken from the vat of each experimental case and, successively, the technological parameters of processing were monitored and noted on specially prepared technical sheets. This made it possible to verify that the cheesemaking operations performed during the trials were the same. 

At the end of processing, the cheese wheels produced in each experiment were marked and left to ripen in the ripening cell of the social dairy, as normally done for the commercial product. The wheels were taken for analyses at 30 days of ripening.

### 2.2. Analyses

#### 2.2.1. Gross Composition and Fatty Acids

For each cheese sample, fat content was determined by the volumetric Gerber method [36], as described by Formaggioni et al. [37]. Moreover, according to Malacarne et al. [38], crude protein by Kjeldahl, [39], salt (NaCl) by potentiometric titration method [40], ash after muffle calcination at 530 °C, and dry matter after oven drying at 102 °C [41], were determined, from which moisture was calculated.

The determination of total fatty acids and CLA was carried out by gas chromatography, by means of two Association of Official Analytical Chemists (AOAC) standard methods [42,43], after derivatisation of the sample according to AOAC standard [44]. Briefly, lipids were extracted from ground cheese (approximately 1 g) with ether–heptane mixture (rate in volume 1:1) after the addition of sodium sulphate and of 2.5 M sulphuric acid 2.5 M. The fatty acids were separated and determined by capillary chromatography with a Carlo Erba GC 6000 Vega Series gas chromatograph (Carlo Erba Instruments, Milan, Italy), equipped with a fused silica capillary column coated with Polyethylene Glycol (30 m × 0.25 mm) Supelco, SP TM 2330 (Sigma-Aldrich Corporation, Saint Louis, MO, USA). The operating conditions were as follows: programmed column temperature from 45 up to 175 °C (13 °C/minutes); then up to 215 °C (4 °C/minutes); stationary at 215 °C for 35 min; injector and detector temperature: 250 °C; carrier gas: hydrogen; column pressure: 175 kPa. The compounds were identified by standard co-injection and relative retention time to FAME 13:0 (internal standard).

Short chain fatty acids (SCFA) were calculated by adding the compounds from C4 to C11; medium chain fatty acids (MCFA) were calculated by adding the compounds from C12 to C16; long chain fatty acids (LCFA) were calculated by adding the compounds from C17 to C24. The other classes were odd fatty acids (OCFA), saturated fatty acids (SFA), monounsaturated fatty acids (MUFA) and polyunsaturated fatty acids (PUFA). Unsaturated fatty acids (UFA) derived from the sum of MUFA and PUFA.

#### 2.2.2. Sensory Analysis

The sensory analysis was only performed on the samples from the winter experimentation. The Triangular test method was adopted [45], which is useful to compare two samples with even small differences. It consisted in presenting three samples to the tasters, two of which were identical: the taster was asked to identify the different samples, and the choice was forced. The comparison between the number of correct and incorrect choices provided the test result. The panel was composed of 40 members who were experts in mountain dairy products.

In order to avoid errors due to the tasting sequence, the sampling plan provided for random distribution of any of the six possible combinations to each taster. The samples were identified by different codes for each judge, using three-digit numbers generated by an algorithm randomly. The chance that the taster has to guess the different sample is 33%, regardless of the perceivability of the difference. The data used were the number of total judgments, the number of correct choices, and the level of significance required for the test. The number of correct choices was compared with the significant theoretical minimum number in two-entry probability tables. If the number of correct choices is greater than or equal to the theoretical one, then we can conclude that there is a significant difference between the two types of samples tested at a certain level of significance.

#### 2.2.3. Volatile Organic Compound (VOC) analysis

Cheese volatile organic compound (VOC) analysis was determined by means of a Head-Space Solid Phase Micro Extraction module (Combi-Pal automated sampler CTC Analytics, Zwingen, Switzerland) equipped with DVB/CAR/PDMS 50/30 μm fiber (Supelco, Bellefonte in Centre, 16823, Pennsylvania, USA) and coupled to a gas chromatograph-mass spectrometer (6890N/5973N Agilent Technologies, Inc., Wilmington, DE, USA). Two and a half grams of cheese were put in a 20 mL head-space glass bottle sealed with a PTFE-silicone septum. Operating conditions: 10 min at 50 °C at 250 rpm; fiber exposition, at 50 °C for 40 min; desorption directly in the injection port of the GC at 260 °C for 10 min. GC column: Zebron ZB-WAX plus (60m × 0.25mm × 10.25 μm, Phenomenex, Torrance, CA, USA) with the following separation conditions: carrier gas helium, in constant flow mode at 1.2 mL/min; oven temperature at 45 °C (10 min), then rising to 150 °C at 5 °C/min, then to 222 °C at 12 °C/min (13 min). Acquisition was performed in electronic impact mode. Transfer line at 280 °C, ion source at 230 °C, quadrupole at 150 °C. Further details are described in Battelli et al. [46]. The mass range used was 39–220 amu. The volatile compounds were identified using the Wiley 7n-1 MS library of Agilent MSD ChemStation® software (Agilent Technologies Inc.). Confirmation of the identity of the volatile compounds was achieved by comparing the GC retention indices and mass spectra of individual components with those of authentic reference compounds injected under the same operating conditions. Data are expressed as arbitrary units of the area of the quant ion of each compound.

### 2.3. Statistical Analysis

The significance of the differences between seasons and between cheese-factories was tested by analysis of variance, using the general linear model procedure of SPSS (IBM SPSS Statics 23, Armonk, New York, NY, USA), according to the following univariate model:

Y_i_ = µ + β_i_ + ε_i_, (1)

where: Y_i_ = dependent variable; µ = overall mean; β_i_ = effect considered in each comparison: housing (summer period only), three levels: stall (S), valley pasture (P), mountain pasture (A); cow feeding (winter only), two levels: silage (I), hay (F); ε_i_ = residual error.

## 3. Results and Discussion

### 3.1. Gross Composition of Formaggella della Valle di Scalve

Table 1 shows the gross composition of Formaggella della Valle di Scalve cheese produced during the winter and summer periods. Data are expressed both for 100 g of cheese and 100 g of dry matter. The cheese was produced with full cream milk, and had a fat-to-protein ratio of approximately 1:1. Since, during the summer period, milk contains more fat (data not shown in the table), the cheese produced in this season showed a higher fat content relative to the winter period, expressed both in 100 g of cheese and 100 g of dry matter. Moisture was just below 45%, and the NaCl content was rather low (around 1.5 g/100g of the cheese).

### 3.2. Fatty Acids

#### 3.2.1. Comparison among Cheese Produced during Summer Season

The results for cheese fatty acid profile reflected the data already found for the corresponding milks (data not shown), confirming the differences in the fatty acid profile. These are not only due to the seasonal variation [47,48], but are closely linked to feed factors. In fact, various authors [30,49] have reported a complete transfer of FA from milk to cheese; hence, their profile largely reflected the raw milk from which they were made. Additionally, Dhiman et al. [50] reported that the FA profile of cow milk was not altered in cheese processing, even when cows were fed different diets.

Table 2 shows the percentage distribution of the fatty acids of the cheese at 30 days. In general, the cheese fat produced from stall milk (S) was richer in medium chain fatty acids (MCFA) relative to that produced from valley (P) and mountain pasture (A). In particular, the most abundant MCFA was palmitic acid (C16), which was higher in S relative to P and A; the second most represented was myristic acid (C14), which was higher in S relative to P, and in P relative to A; finally, C12 (lauric), C12:1 (lauroleic) and C13 (tridecanoic) were higher in S than in the P and A samples. On the other hand, long chain fatty acids (LCFA) were higher in P and A cheeses than in S ones. Oleic acid, the most represented LCFA, had a lower concentration in S and P samples relative to A; stearic acid, was lower in S than in P and A cheeses. Cheese from mountain pasture (A) also had more arachidic (C20) acid, while linolenic acid (C18:3) was higher in valley pasture (P).

In general, MUFA, PUFA and, consequently, total unsaturated (UFA), were significantly higher in the cheeses produced from both valley (P) and mountain pasture (A) milk (UFA: 31.13 S vs. 36.55 P and 38.34 A; *p* < 0.001), while SFA showed higher values in stall milk cheeses. 

Carafa et al. [51], for traditional mountain cheeses, reported similar values for myristic acid (9.4 g/100g FA), but lower values for palmitic acid (22.6 g/100g FA), and stearic acid (8.1 g/100g FA), while oleic acid (22.5 g/100g FA) is consistent with value registered for P, but lower than of A, in the present research. In the literature, differences in milk FA profiles from cows fed pasture or hay and concentrates are well known. Various authors [49,52] have found higher amounts of short-, and medium-chain FAs, C16:0 and total saturated FAs (SFA) in indoor cows’ milk. 

In contrast, all the mono-unsaturated FAs (MUFA), all the conjugated linoleic acid (CLA) isomers, and the total poly-unsaturated FAs (PUFAs), were higher in pasture-based milk and cheese. The mechanism is well known: compared with indoor cows’ milk, the content of de novo ( <16 C) FA slightly decreased in pasture-based milk. A general reduction of de novo FA occurs in milk from grazing cows because high levels of dietary PUFA from pasture can compete with de novo fatty acids for esterification in the mammary gland, and thus determine a decrease in the synthesis of short- and medium-chain fatty acids [49].

Moreover, a negative energy balance may occur in lactating cows on pasture, thus reducing the synthesis of short- and medium-chain FA in the mammary gland [27,53]. In agreement with several studies, i.e., those reported by Collomb et al. [54] in a review, FA of mixed origin (C16:0 and C16:1) were lower in pasture-based milk, while C18 was higher in pasture milk.

Esposito et al. [49] observed that, consequently, unsaturated FA content increased to the detriment of SFA content in the cheese. This can result in a more favourable FA composition in cheese from grazing cows. Therefore, a wider use of pasture may be promoted in order to accentuate this positive feature; this is important from the point of view of promoting mountain dairy products. From a nutritional and health point of view, it is important to reduce the level of saturated fatty acids relative to unsaturated ones in the diet [55].

In the present study, conjugated linoleic acids, and in particular rumenic acid (and its precursor vaccenic acid t-11) were more represented in the P and A samples. Values for total CLAs were in agreement with the data reported by Carafa et al. [51] for traditional mountain cheeses (but the concentration of vaccenic acid of 2.4 g/100g FA was higher than that found in the present experiment). A partial agreement with the results of Revello Chion et al. [30] was also found: these authors registered, in Toma Piemontese cheese (a semi-hard cheese like Formaggella della Valle di Scalve), average values of 2.09% and 0.81% total FA in summer and winter, respectively. Seasonal variation is mainly due to animal feeding: in fact, as in the present study, also in the research of Revello Chion et al. [30] cows in winter were housed indoors and fed hay and concentrates, whereas in summer were fed natural pasture. Relative to the values found by these authors, the CLA values registered in the present study for valley pasture, and particularly those of mountain pasture (A), were perceptibly lower, and this is probably due to the different compositions of the pastures. Our values are in agreement with those reported by Lobos-Ortega et al. [56] in a study on the different CLA value of cheeses from three different species. 

The variation of CLA content in milk has been associated with several factors, such as diet of cows, breed and stage of lactation, but diet is the most important variation factor. The difference between cows reared indoor and cows fed pasture has been reported by various authors: all studies indicated higher CLA values in the case of pasture [52,57,58,59,60]. Di Grigoli et al. [61], in their study on Caciocavallo, an Italian ripened pasta filata cheese, reported that the utilization of pasture, relative to hay-based feeding, almost doubled the level of C18:2 *trans*-10,*cis*-12 (CLA). Coppa et al. [57] also reported that all the conjugated linoleic acid (CLA) isomers were lower in the milk of cows reared indoors. Esposito et al. [49] and Chilliard et al. [27] found an increase in the unsaturated fraction and CLA contents in dairy products derived from grazing systems. Prandini et al. [62], in Grana Padano cheese, reported that CLA concentration was higher in mountain cheese (mainly Trentin Grana, a particular Grana Padano for which animals are bred in the mountains with altitude > 800 m, and silages are forbidden) (6.52 and 9.47 mg/g fat in spring and summer respectively) compared with lowland cheese (5.29 and 5.75 mg/g fat in spring and summer respectively), with values increasing from spring to summer in all analyzed samples, but especially in mountain Grana Padano. 

As described by many authors, [62,63,64], in milk from ruminants there is an amount of rumenic acid (*cis*-9, *trans*-11 CLA) due to incomplete biohydrogenation of polyunsaturated fatty acids (PUFA), specially linoleic and α-linolenic acids, in the rumen, and then from desaturation of vaccenic acid in the mammary gland via Δ9-desaturase [65]. In particular, up to 99% of α-linolenic acid and linoleic acid consumed by cows is biohydrogenated in the rumen, with vaccenic acid being a main derivative [66]. Vaccenic acid is then partly desaturated to CLA in the mammary gland, explaining the elevated CLA content in milk from predominantly-grass-fed cows [63]. In fact, when fed indoors, cows have no access to grass, which is rich in the linoleic and α-linolenic acids involved in the synthesis of CLA [59]; a higher dietary intake of α-linolenic acid should consequently lead to a higher amount of CLA in milk.

In fact, the value of α-linolenic acid in the present study is higher in P cheese, also relative to A cheese; moreover, although the difference is not significant, our CLA value for valley pasture tends to be higher than that for mountain (alpine) pasture. This result is supported by Leiber et al. [63] who reported that lowland pasture contained almost twice as much α-linolenic acid, resulting in 25% more CLA in milk relative to cows grazing alpine pasture. Various authors have suggested that fresh grass promotes the synthesis of CLA through a greater activity of Δ^9^-desaturase in the udder [67,68]. Moreover, the high concentrations of soluble fiber and fermentable sugars in fresh grass can create an environment in the gastrointestinal tract of ruminants, without lowering the pH, that is favorable for the growth of the bacteria responsible for synthesizing CLA and the production of vaccenic acid [50].

Omega 3 fatty acids, and in particular α-linolenic acid, were in the present research more represented in cheese from valley pasture (P) milk relative to cheese from stall (S) milk (A milk being in an intermediate position). In contrast, Omega 6 fatty acid percentages were not statistically different among the three experimental situations. The higher content of Omega 3 FA in the cheese from pasture is confirmed by Cozzi et al. [69], who in mountain pasture cheese found a lower content of short chain fatty acids and C16:0, and an increase in unsaturated fatty acids and Omega 3 fatty acids. It is well-known that milk fatty acid composition is affected by the cow’s feeding plan and particularly by the amount and the quality of the forage included in the diet [70]. Additionally, Zeppa et al. [71], in the production of Ossolano cheese (an Italian semi-hard cheese), in cows fed exclusively green forage in mountain pastures, found an increase in Omega 3 fatty acids and a decrease in the n-6/n-3 ratio, with a very important nutritional effect. In fact, Omega 3 polyunsaturated fatty acids are recognized as playing an essential role in human health and are particularly important for the proper functioning of the brain, the heart and the retina of the eye [72].

#### 3.2.2. Comparison between Cheeses Produced in Winter Season

The fatty acid profile (Table 3) showed numerous differences between the two experimental groups. In particular, the cheeses in case I (silage) presented higher percentages of myristic (C14) and myristoleic (C14:1) acids and a lower percentage of C15, C17, C17:1, stearic acid and arachidic acid. A statistically significant difference was found between these two cases for SFA and MUFA, as well as for MCFA and LCFA, while total PUFA were higher for I than for F. Moreover, for I, the percentages of omega 6 were higher (in particular for linoleic acid) and the percentages of CLA (rumenic acid) and also of its metabolic precursor, vaccenic acid, were lower. 

There are few studies on the effect of hay or silage cow feeding on the cheese fatty acid profile, and most of them are related to maize silage [73], while another considers vetch hay vs. vetch silage in sheep milk and cheese [74]. The result that PUFA are higher in hay silage (haylage) than in hay is consistent with the study of Schingoethe et al. [75] that found the same result, explaining it as a trend toward increased unsaturation for feeding fermented forages [75]. On the contrary, other authors [74] found higher values of PUFA in the hay group than in the silage group, but the silage is not the same (in this case it is vetch silage, and the research is on sheep milk and cheese).

The result for SFA, which had no significant variations, is consistent with results of Renes et al. [74]. The higher values of CLA and vaccenic acid found in hay vs. silage in the present research is confirmed by Segato et al. [73], although these authors consider maize silage instead of hay silage.

In the present research, the concentration of elaidic (trans) acid was higher in type I cheeses. Elaidic acid is a trans fatty acid associated with an increased risk of coronary heart disease, which can deleteriously affect lipoproteins by increasing LDL and decreasing HDL [76]. 

Odd fatty acids, in the present research, were more represented in cheese F. They are an index of a higher ruminal activity, as attested also by the higher contents of vaccenic and rumenic acids in the F thesis. Odd fatty acids have, by some authors [60,73], been associated with beneficial effects on human health [77]; in synthesis, except for the lower contents of PUFA and Omega 6 fatty acids, hay cow feeding without silage results in an overall more favourable fatty acid profile for human health.

### 3.3. Sensory and VOC Analyses: Comparison between Cheeses Obtained from Cows Fed Hay and Silage at 30 Days of Ripening

In order to evaluate any possible differences in taste and flavour between hay and silage production, cheeses at 30 days ripening were submitted to both sensorial and VOC analyses. The results of the triangular test carried out on the cheeses produced on 5 different days (two different winters), show that the cheese samples were judged significantly different in only 2 out of 5 days of production (Table 4). Such “insignificance” can also be due to the difficult standardisation of cheesemaking procedures in mountain environments, which in some cases can prevail over differences in the raw material used.

**Figure 1:**
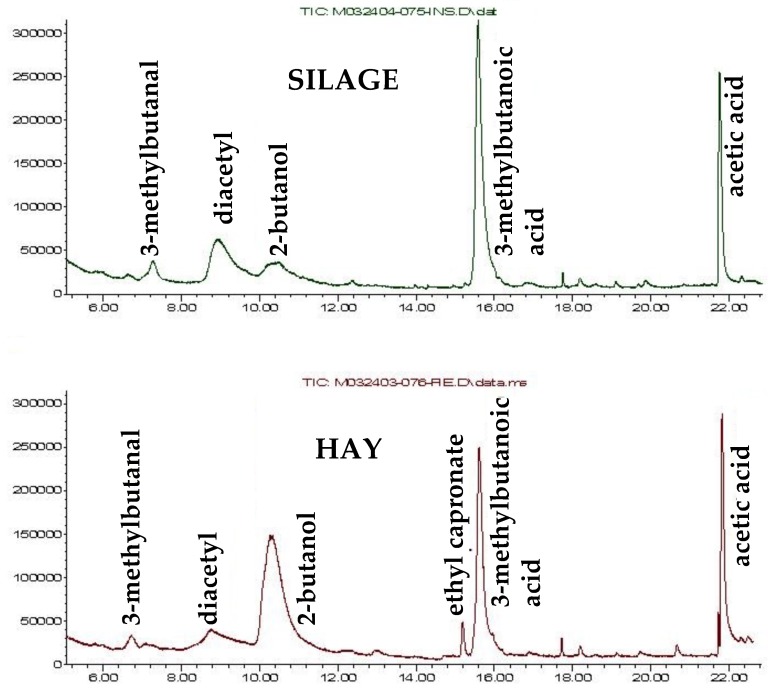
Chromatogram of total ionic current from dynamic headspace coupled for the two cheese typologies.

After choosing the “different” sample, the tasters were also asked to make a preference judgment, and to motivate it, and both hay and silage cheeses were equally preferred. It should be noted that in this kind of cheese (artisanal mountain cheese), personal tastes can differ dramatically, and what is a pleasant note for one taster, can be a defect for another. Apart from the appreciation, the recurring descriptors were “sweet”, “spicy”, “savoury”, and “fruity”. The first three attributes are manly perceived by taste and are connected to water or fat-soluble compounds released during chewing, such as free amino acids and short chain free fatty acids. On the other hand, fruity perception is due to volatile substances, mainly esters, but also some alcohols, aldehydes and ketones [78,79]. In this regard, the evaluation of the volatile profile of the cheeses obtained by the SPME-GC-MS analysis of the sample headspace (data not reported) confirms sensorial analysis. Even though further investigations must be made, the total ion chromatograms reported in Figure 1 suggest a possible consistency between testers’ assessments and the instrumental data. The chromatogram for a “hay” sample judged as “sweet” and “fruity”, compared to the corresponding “silage”, shows higher levels of 2-butanol (fruity aroma) and ethyl capronate (banana, pineapple smell), which can both explain the higher fruity and sweet perceptions [80].

Verdier-Metz et al. [12], even though without statistically significant differences, also found that the cheeses produced with milk from cows fed silage seem to be less sticky and more bitter than the cheeses produced with milk from cows fed hay. The more pronounced bitterness of “silage” cheeses could be due to quicker ripening [81] and to proteolysis tending to produce hydrophobic peptides, which are generally bitter [82].

## 4. Conclusions

In conclusion, the experimentation demonstrated that the fat of the cheese obtained from stall milk was richer in medium-chain fatty acids. On the other hand, long-chain fatty acids were higher in the cheeses produced from pasture milk. Oleic acid was higher in the case of mountain pasture milk, whereas omega 3 fatty acids were more present in the cheeses produced on low altitude pasture. The content of unsaturated fatty acids and CLAs was always positively influenced by pasture. 

The results of the sensory analysis, conducted on winter samples, show that the tasters were not always able to find significant differences. In general, cheeses produced with hay feeding were judged as “sweeter” than cheeses produced with silage feeding. The volatile profile of the F cheese presented higher levels of 2-butanol (fruity aroma) and ethyl capronate (smell of banana, pineapple), which can explain both the higher fruity and sweet perceptions. The results of a triangular test were compared with “cheese volatile organic compound (VOC) analysis”; the obtained chromatogram suggests a possible consistency between the assessments of the tasters and the instrumental data.

## Figures and Tables

**Table 1:** Average gross composition of Formaggella della Valle di Scalve cheese at 30 days’ ripening produced during the winter and summer periods.

		Gross Composition on 100 of Cheese		Gross Composition on 100g of Dry Matter	
		Mean	SD	Mean	SD
Winter:					
Moisture	g/100g	42.48	3.56	-	-
Protein	g/100g	25.60	0.80	44.64	3.16
Fat	g/100g	25.58	4.26	44.25	4.98
Ash	g/100g	4.04	0.16	7.05	0.71
NaCl	g/100g	1.61	0.21	2.82	0.50
Summer:					
Moisture	g/100g	44.63	2.88	-	-
Protein	g/100g	24.35	1.70	44.08	3.91
Fat	g/100g	27.24	2.95	49.12	1.27
Ash	g/100g	3.59	0.63	6.49	1.08
NaCl	g/100g	1.47	0.34	2,65	0.59

**Table 2:** Percent distribution of FA of 30 days ripening cheese (comparison between milk produced in stall, valley pasture and mountain pasture). Mean ± SD. a,b,c, differ for *p* < 0.05. NS, *p* > 0.05; * *p ≤* 0.05; ** *p ≤* 0.01; *** *p ≤* 0.001.

		Stall		Valley Pasture		Mountain Pasture		*p*
Number of Observations		5		5		5		
C4—Butiric	%	2.28 ± 1.15		2.25± 0.48		2.77 ± 0.92		NS
C6—Capronic	%	1.74 ± 0.86		1.55 ± 0.26		1.49 ± 0.32		NS
C8—Caprilic	%	1.26 ± 0.52		1.04 ± 0.14		0.86 ± 0.14		NS
C10—Caprinic	%	3.28 ± 0.97	b	2.43 ± 0.30	ab	1.83 ± 0.17	a	*
C10:1—Decenoic	%	0.32 ± 0.12		0.26 ± 0.05		0.21 ± 0.04		NS
C12—Lauric	%	3.92 ± 0.69	b	2.93 ± 0.33	a	2.30 ± 0.14	a	**
C12:1—Lauroleic	%	0.12 ± 0.02	b	0.09 ± 0.02	a	0.07 ± 0.02	a	*
C13—Tridecanoic	%	0.14 ± 0.02	b	0.10 ± 0.02	a	0.10 ± 0.03	ab	*
C14—Myristic	%	12.79 ± 0.95	c	10.91 ± 0.73	b	9.57 ± 0.33	a	***
C14:1—Myristoleic	%	1.02 ± 0.12	b	0.90 ± 0.14	ab	0.80 ± 0.06	a	*
C15—Pentadecanoic	%	1.31 ± 0.06		1.37 ± 0.03		1.28 ± 0.12		NS
C16—Palmitic	%	30.58 ± 1.86	b	27.44 ± 1.12	a	26.64 ± 2.29	a	*
C16:1—Palmitoleic	%	1.29 ± 0.04		1.28 ± 0.07		1.26 ± 0.11		NS
C17—Eptadecanoic	%	0.82 ± 0.04		0.85 ± 0.07		0.87 ± 0.08		NS
C17:1—Eptadecenoic	%	0.25 ± 0.05		0.25 ± 0.04		0.28 ± 0.03		NS
C18—Stearic	%	10.31 ± 1.21	a	12.15 ± 0.98	b	13.57 ± 0.53	b	**
C18:1—Elaidic t-9	%	0.50 ± 0.13		0.70 ± 0.26		0.73 ± 0.33		NS
C18:1—Vaccenic t-11	%	1.89 ± 0.40	a	4.29 ± 0.48	b	3.49 ± 0.78	b	***
C18:1—Oleic	%	20.63 ± 2.54	a	22.70 ± 0.80	a	26.03 ± 1.25	b	**
C18:1—Vaccenic c-11	%	0.41 ± 0.07		0.41 ± 0.05		0.46 ± 0.05		NS
C18:2—Linoelaidic t-6	%	0.20 ± 0.02		0.23 ± 0.01		0.21 ± 0.03		NS
C18:2—Linoleic	%	2.62 ± 0.43		2.26 ± 0.17		2.19 ± 0.69		NS
C20—Arachidic	%	0.18 ± 0.03	a	0.20 ± 0.02	a	0.25 ± 0.05	b	*
C20:1—Eicosenoic	%	0.06 ± 0.01	ab	0.04 ± 0.01	a	0.07 ± 0.01	b	*
C18:3—α-linolenic	%	0.78 ± 0.07	a	1.02 ± 0.07	b	0.83 ± 0.21	a	*
C18:2—Rumenic c9,t11 CLA	%	0.78 ± 0.26	a	1.82 ± 0.18	b	1.49 ± 0.26	b	***
C18:2—t10-c12 CLA	%	0.02 ± 0.03		0.04 ± 0.06		0.03 ± 0.03		NS
SCFA—short chain FA	%	8.97 ± 3.49		7.58 ± 1.04		7.18 ± 1.44		NS
MCFA—middle chain FA	%	51.17 ± 2.26	b	45.01 ± 2.02	a	42.02 ± 2.52	a	***
LCFA—long chain FA	%	39.84 ± 4.10	a	47.37 ± 2.29	b	50.82 ± 1.30	b	***
OCFA—odd FA	%	2.69 ± 0.20		2.67 ± 0.23		2.61 ± 0.23		NS
MUFA—monounsaturated FA	%	26.47 ± 2.52	a	30.93 ± 0.99	b	33.38 ± 1.57	b	***
PUFA—polyunsaturated FA	%	4.66 ± 0.42	a	5.62 ± 0.25	b	4.96 ± 0.78	ab	*
UFA—unsaturated FA	%	31.13 ± 2.86	a	36.55 ± 1.11	b	38.34 ± 0.94	b	***
SFA—saturated FA	%	68.85 ± 2.84	b	63.41 ± 1.10	a	61.68 ± 0.91	a	***
CLA (conjug. linoleic acids)	%	0.80 ± 0.28	a	1.86 ± 0.21	b	1.52 ± 0.27	b	***
Omega 3 FA	%	0.83 ± 0.12	a	1.09 ± 0.14	b	0.86 ± 0.22	ab	*
Omega 6 FA	%	2.83 ± 0.49		2.44 ± 0.21		2.37 ± 0.77		NS

**Table 3:** Percentage distribution of the fatty acids of the cheese at 30 days ripening relative to the comparison of the winter period between milk produced from cows fed silage and milk produced from cows fed hay. Mean ± SD. a, b, differ for *p* < 0.05. NS, *p* > 0.05; * *p* ≤ 0.05; ** *p* ≤ 0.01.

		Silage (I)		Hay (F)		*p*
Number of Observations		3		3		
C4—Butiric	%	2.85 ± 1.33		2.27 ± 0.20		NS
C6—Capronic	%	2.19 ± 1.14		1.58 ± 0.36		NS
C8—Caprilic	%	1.52 ± 0.72		1.04 ± 0.25		NS
C10—Caprinic	%	3.75 ± 1.35		2.53 ± 0.44		NS
C10:1—Decenoic	%	0.41 ± 0.14	b	0.26 ± 0.01	a	*
C12—Lauric	%	4.33 ± 0.90		3.21 ± 0.24		NS
C12:1—Lauroleic	%	0.14 ± 0.01		0.11 ± 0.03		NS
C13—Tridecanoic	%	0.14 ± 0.01		0.12 ± 0.02		NS
C14—Myristic	%	13.22 ± 0.82	b	12.18 ± 0.31	a	*
C14:1—Myristoleic	%	1.15 ± 0.09	b	0.94 ± 0.14	a	*
C15—Pentadecanoic	%	1.30 ± 0.05	a	1.42 ± 0.08	b	*
C16—Palmitic	%	31.29 ± 2.64		32.99 ± 2.99		NS
C16:1—Palmitoleic	%	1.31 ± 0.02		1.52 ± 0.24		NS
C17—Eptadecanoic	%	0.79 ± 0.10	a	0.96 ± 0.02	b	*
C17:1—Eptadecenoic	%	0.23 ± 0.08	a	0.35 ± 0.06	b	*
C18—Stearic	%	9.02 ± 1.15	a	10.81 ± 0.96	b	*
C18:1—Elaidic t-9	%	0.68 ± 0.06	b	0.48 ± 0.03	a	**
C18:1—Vaccenic t-11	%	1.28 ± 0.24	a	1.83 ± 0.17	b	**
C18:1—Oleic	%	19.45 ± 1.74		20.87 ± 0.35		NS
C18:1—Vaccenic c-11	%	0.40 ± 0.08		0.46 ± 0.02		NS
C18:2—Linoelaidic t-6	%	0.20 ± 0.01	a	0.25 ± 0.02	b	**
C18:2—Linoleic	%	2.55 ± 0.20	b	1.87 ± 0.17	a	**
C20—Arachidic	%	0.16 ± 0.03	a	0.21 ± 0.01	b	*
C18:3—α-linolenic	%	0.87 ± 0.07		0.76 ± 0.08		NS
C18:2—Rumenic c9,t11 CLA	%	0.65 ± 0.08	a	0.87 ± 0.08	b	**
C18:2—t10-c12 CLA	%	0.01 ± 0.00		0.02 ± 0.01		NS
SCFA—short chain FA	%	10.70 ± 4.68		7.67 ± 1.26		NS
MCFA—middle chain FA	%	52.87 ± 0.89		52.47 ± 2.97		NS
LCFA—long chain FA	%	36.39 ± 3.72		39.84 ± 1.69		NS
OCFA—odd FA	%	2.45 ± 0.23	a	2.85 ± 0.06	b	*
MUFA—monounsaturated FA	%	25.03 ± 1.84		26.80 ± 0.17		NS
PUFA—polyunsaturated FA	%	4.40 ± 0.37	b	3.88 ± 0.17	a	*
UFA—unsaturated FA	%	29.43 ± 2.21		30.68 ± 0.35		NS
SFA—saturated FA	%	70.53 ± 2.27		69.29 ± 0.36		NS
CLA (conjug. linoleic acids)	%	0.66 ± 0.08	a	0.89 ± 0.07	b	**
Omega 3 FA	%	0.87 ± 0.07		0.76 ± 0.08		NS
Omega 6 FA	%	2.68 ± 0.23	b	1.99 ± 0.14	a	**

**Table 4:** Results of the triangular test made on cheese samples at 30 days’ ripening.

Day of Cheesemaking	Difference ^1^
**1 February**	*
**16 March**	NS
**6 January**	***
**24 February**	NS
**26 March**	NS

^1^ NS, not significantly different; * *p* ≤ 0.05; *** *p* ≤ 0.001.
